# Case Report: COVID-19 exacerbates acute lower limb ischemia in patients with popliteal artery entrapment syndrome

**DOI:** 10.3389/fcvm.2024.1329863

**Published:** 2024-02-02

**Authors:** Li Bo, Du Xiaojiong

**Affiliations:** Department of Vascular Surgery, West China Hospital, Sichuan University, Chengdu, China

**Keywords:** case report, non-traumatic lower limb ischemic diseases, young adults, popliteal artery entrapment syndrome (PAES), COVID-19

## Abstract

Non-traumatic lower limb ischemic diseases are extremely rare among young people. Clinically, they are mainly seen in the form of popliteal artery entrapment syndrome (PAES). In addition, with the prevalence of COVID-19 infection, more and more studies report that COVID-19 infection may lead to arteriovenous thrombosis, which could cause lower limb ischemia. This case reported that a 31-year-old male amateur football player who developed intermittent claudication after recovering from COVID-19. After 2 months of consultation, he was ultimately diagnosed with PAES. As is well known, PAES is mostly caused by long-term compression of the popliteal artery by abnormal anatomical structures, resulting in thickening of the vascular outer membrane and progression of the disease until intimal damage and thrombosis, leading to lower limb ischemia. During the progression of the disease, there may be multiple factors that accelerate its progression. Therefore, combined with the patient's clinical history and related studies on confirmed thrombosis caused by COVID-19, we can infer that COVID-19 could accelerate the occurrence of PAES.

## Introduction

Popliteal artery entrapment syndrome (PAES) is a rare clinical ischemic disease of the lower limbs, mainly caused by various reasons such as popliteal artery compression and stenosis and secondary thrombosis caused by an injury of the vascular endothelium due to exercise and other factors (1–3). It is more common among young people, especially sports professionals and enthusiasts (3). The main manifestation is progressive motor pain in the lower limbs, and the combination of high-risk factors such as infection and smoking for thrombosis will exacerbate lower limb ischemia. If not diagnosed and treated appropriately, it may lead to disability and even serious consequences, such as amputation (4). This disease is easily confused with lower extremity arterial ischemic diseases such as lower extremity arterial thromboangiitis obliterans, lower extremity arterial embolism, and lower extremity atherosclerosis obliterans (5, 6). Therefore, clinical diagnosis is difficult and prone to misdiagnosis, which can affect treatment decisions and clinical prognosis. At present, there are many reports on cases of PAES, but there is no report on cases of acute exacerbation of lower limb ischemia induced by COVID-19.

## Case description

**Figure 1:**
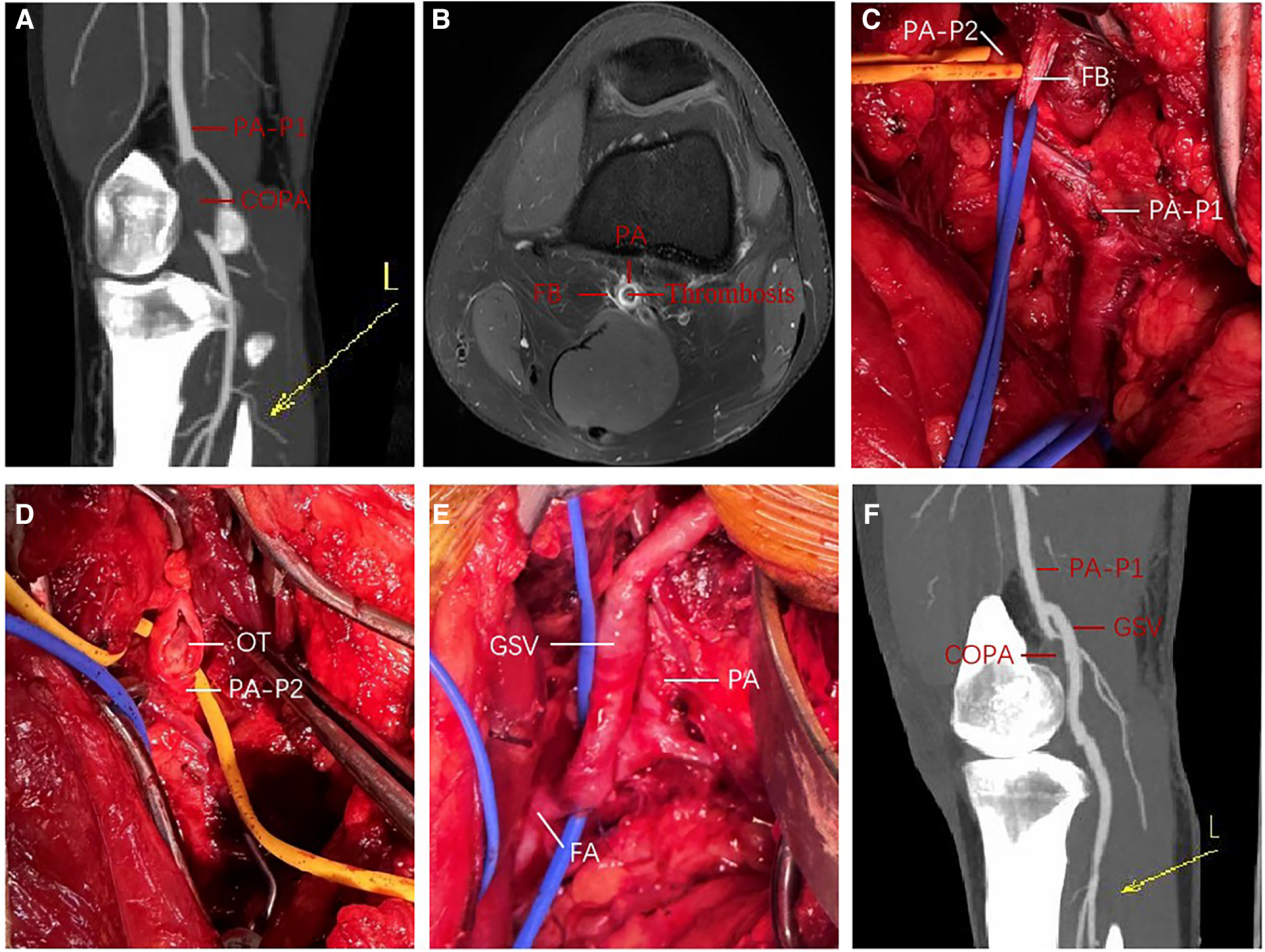
Perioperative findings in a young man with PAES; (**A**) preoperative 3D contrast-enhanced CTA of left lower extremity; (**B**) preoperative enhanced MRI T1W imaging of left knee joint; (**C**,**E**) intraoperative exploration and left popliteal artery autologous saphenous vein bypass; (**F**) 3D contrast-enhanced CTA of left lower extremity at 3 months after operation. COPA, completely occluded popliteal artery; CTA, computed tomography angiography; FA, femoral artery; FB, fibrous band; GSV, great saphenous vein; L, left lower extremity; MRI, magnetic resonance imaging; OT, organic thrombus; PA, popliteal artery; PA-P1, the P1 segment of the popliteal artery; PA-P2, the P2 segment of the popliteal artery; PAES, popliteal artery entrapment syndrome; 3D, three-dimensional.

**Figure 2:**
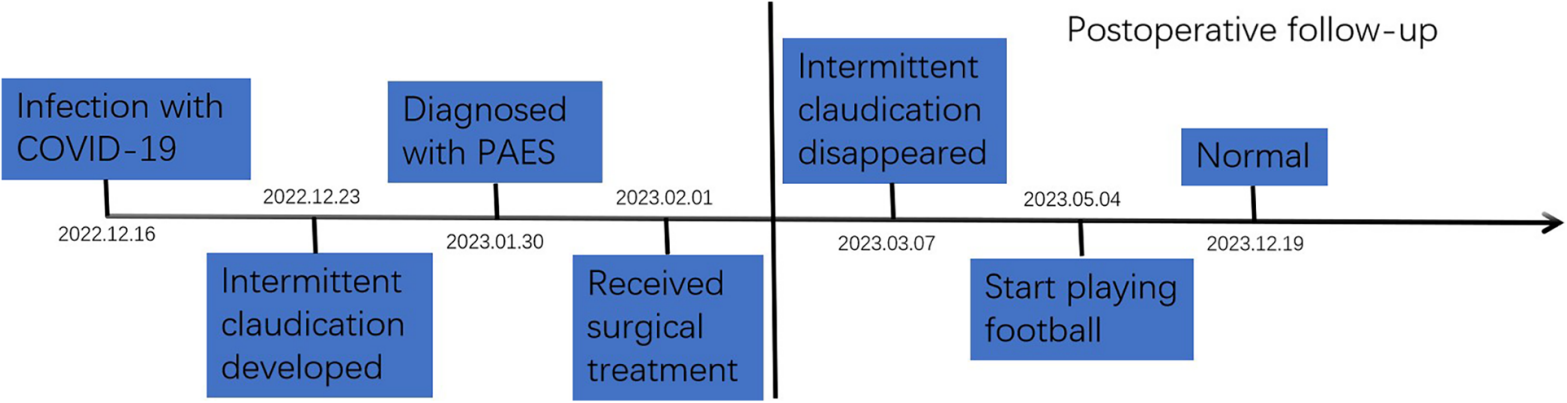
The timeline diagram of the diagnosis, treatment, and follow-up of the patient with PAES. PAES, popliteal artery entrapment syndrome.

A 31-year-old male amateur football player who took part in football 1 week after recovering from COVID-19 developed intermittent claudication, accompanied by pain and discomfort of the left lower limb. The symptoms of claudication did not subside for 2 months, and the pulsation of the left dorsal foot artery was decreased. During this period, he had been traveling to many hospitals without a clear diagnosis. After admission to our hospital, lower limb computed tomography angiography (CTA) indicated thrombosis in the P2 segment of the left popliteal artery, with complete occlusion of the lumen (Figure 1A). Magnetic resonance imaging (MRI) of the left knee joint indicated the filling defect in the P2 segment of the left popliteal artery was considered to be possibly due to thrombosis, and the superficial surface of the P2 segment of the popliteal artery was suspected to be crossed by an abnormal ribbon of fibers (Figure 1B). Furthermore, functional color doppler ultrasound revealed that the bilateral popliteal arteries showed compression and stenosis of the lumen in the extended position. We performed left popliteal artery lesion resection and autologous saphenous vein bypass surgery for the patient. Exploring the popliteal fossa confirmed that the strip-like ligament passed through the surface of the popliteal artery, and the affected popliteal artery showed a strip-like change without pulsation (Figure 1C). The fibrous strips on the surface of the popliteal artery were severed and loosened, and an organic thrombus was found within the popliteal artery (Figure 1D). The bypass popliteal artery was well filled and pulsated after surgery (Figure 1E). After 3 months of anticoagulation therapy with 20 mg rivasaban tablets once a day, the patient started playing football without symptoms in the lower limbs. Moreover, the lower limb CTA showed that a patent great saphenous vein bypassed the left popliteal artery (Figure 1F). Figure 2 shows the timeline diagram of the diagnosis, treatment, and follow-up of the patient.

## Discussion

PAES is a rare disease that often causes intermittent claudication of the lower limbs and is common among athletes. Its incidence rate is 0.6%–3.5% (3, 4). PAES is based on congenital anatomical abnormalities, such as abnormal development of the popliteal fossa structure that compresses the popliteal artery. These cases are classified according to the Love and Whelan classification modified by Rich (7). As it was found that the fibrous cord had collapsed through the popliteal artery, this case was type IV. The preoperative diagnosis of PAES mainly relies on imaging examinations such as lower limb ultrasound, CTA, and, especially, MRI, due to its advantages in soft tissue imaging (8, 9). Even so, the imaging manifestations of the soft tissue around the compressed popliteal artery are still difficult to detect (8). Therefore, it is easily misdiagnosed as lower limb arterial thromboangiitis obliterans, lower limb arterial embolism, and other ischemic diseases (5, 6). Furthermore, certain factors may lead to the worsening of PAES ischemia. These include blood hypercoagulability and vascular endothelial damage (10). Interestingly, this case reported that the patient developed intermittent claudication 1 week after COVID-19 infection and had no symptoms before that. At present, some studies have reported that patients with COVID-19 have arteriovenous thrombosis (11–14). COVID-19 causes endothelial cell damage, exposes collagen, causes coagulation activation, and then induces thrombosis (11–14). Generally speaking, the abnormal fibrous band crossing the superficial surface of the popliteal artery in patients is innate. In addition, it is well known that PAES is often caused by long-term compression of the popliteal artery by abnormal anatomical structures, resulting in thickening of the outer membrane of the blood vessels and progression of the disease until intimal damage and thrombosis, leading to lower limb ischemia. During the progression of the disease, there may be multiple factors that accelerate the progression of the disease. Therefore, combined with the patient's clinical history and related studies on confirmed thrombosis caused by COVID-19, we can infer that COVID-19 could accelerate the occurrence of PAES. Of course, these are just speculations, and there is currently no relevant laboratory test to support them. Ultimately, large-scale clinical studies are needed for validation.

When patients have early symptoms of lower limb pain, it is necessary to actively perform relevant examinations and perform a clear diagnosis to avoid delaying treatment. Anticoagulants and vasodilators are the conventional therapy. Furthermore, open surgery and endovascular intervention are the fundamental therapies to cure patients. The key to treating PAES lies in relieving popliteal artery compression and restoring the blood supply to the affected limb. Clinically, for patients with acute ischemia whose disease duration is less than 2 weeks, femoral artery incision thrombectomy or catheterization thrombolysis of the affected limb can be selected to restore the blood supply of the lower limb (15, 16). Furthermore, for patients with a disease duration of more than 2 weeks, thrombectomy, venous patch arterioplasty, and autologous/artificial vessel bypass surgery can be adopted to restore the blood supply of the affected lower limb (17, 18). Moreover, based on the current literature results, vascular bypass surgery is recommended as a priority for blood flow reconstruction (18). Thus, popliteal artery bypass surgery may be the best treatment for PAES (19, 20).

## Conclusion

PAES is the most common non-traumatic lower limb ischemic disease in young people, and its diagnosis relies on CTA/MRI and clinical history during exercise. All patients with clear PAES should undergo surgical treatment. COVID-19 may increase the risk of arteriovenous thrombosis, which may further aggravate the acute ischemia of lower limbs in people with PAES. Thus, these people tend to be given preventive anticoagulant treatment to avoid serious complications such as amputation. However, due to the limitations of case reports, this still needs further research with large samples for further confirmation.

## Data Availability

The datasets presented in this study can be found in online repositories. The names of the repository/repositories and accession number(s) can be found in the article/Supplementary Material.
